# Degraded inferior colliculus responses to complex sounds in prenatally exposed VPA rats

**DOI:** 10.21203/rs.3.rs-3168097/v1

**Published:** 2023-07-31

**Authors:** Yuko Tamaoki, Varun Pasapula, Collin Chandler, Michael S. Borland, Olayinka I. Olajubutu, Liza S. Tharakan, Crystal T. Engineer

**Affiliations:** The University of Texas at Dallas School of Behavioral and Brain Sciences; The University of Texas at Dallas School of Behavioral and Brain Sciences; The University of Texas at Dallas School of Behavioral and Brain Sciences; The University of Texas at Dallas School of Behavioral and Brain Sciences; The University of Texas at Dallas School of Behavioral and Brain Sciences; The University of Texas at Dallas School of Behavioral and Brain Sciences; The University of Texas at Dallas School of Behavioral and Brain Sciences

**Keywords:** Autism, Auditory Processing, Inferior Colliculus, Valproic Acid

## Abstract

**Background:**

Individuals with autism spectrum disorders (ASD) often exhibit altered sensory processing and deficits in language development. Prenatal exposure to valproic acid (VPA) increases the risk for ASD and impairs both receptive and expressive language. Like individuals with ASD, rodents prenatally exposed to VPA exhibit degraded auditory cortical processing and abnormal neural activity to sounds. Disrupted neuronal morphology has been documented in earlier processing areas of the auditory pathway in VPA-exposed rodents, but there are no studies documenting early auditory pathway physiology. Therefore, the objective of this study is to characterize inferior colliculus (IC) responses to different sounds in rats prenatally exposed to VPA compared to saline-exposed rats.

**Methods:**

Neural recordings from the inferior colliculus were collected in response to tones, speech sounds, and noise burst trains.

**Results:**

Our results indicate that the overall response to speech sounds was degraded in VPA-exposed rats compared saline-exposed controls, but responses to tones and noise burst trains were unaltered.

**Conclusions:**

These results are consistent with observations in individuals with autism that neural responses to complex sounds, like speech, are often altered, and lays the foundation for future studies of potential therapeutics to improve auditory processing in the VPA rat model of ASD.

## Introduction

Individuals with autism spectrum disorders (ASD) often exhibit altered sensory processing and deficits in language development. This manifests as a range of auditory processing challenges across individuals, from a heightened sensitivity to sound to a diminished sensitivity to sound. These perceptual changes are accompanied by alterations in the neural response to sound across the auditory pathway [1, 2]. For example, auditory cortex responses to vowel sounds are significantly delayed in verbal children with ASD compared to typically developing children, even further delayed in language impaired children with ASD, and profoundly delayed in minimally verbal and nonverbal children with ASD [3]. Children with ASD benefit from traditional rehabilitation therapies, but many children undergo these time-consuming expensive therapies, and still experience deficits. The development of new techniques to enhance auditory processing in individuals with neurodevelopmental disorders is necessary.

Children who are prenatally exposed to the anticonvulsant medication valproic acid (VPA) have an increased risk for ASD, learning impairments, and developmental delays [4]. Prenatal exposure to VPA can result in ASD symptoms including altered sensory processing and deficits in language development [5–8]. Children prenatally exposed to VPA have impaired receptive and expressive language that is dose dependent: children who were prenatally exposed to larger VPA doses have worse language abilities than children who were exposed to smaller doses [9].

Like humans, rodents prenatally exposed to VPA exhibit degraded auditory processing and abnormal cortical neural activity to sounds. VPA exposed rats are impaired at discriminating between sounds differing in the initial consonant (“dad” vs. “bad”) compared to saline exposed control rats [10]. In the auditory cortex, responses are weaker, delayed, and have disorganized tonotopy in VPA exposed rodents [1, 11, 12]. In subcortical structures, fewer neurons and disrupted neuronal morphology have been documented in the superior olivary complex, lateral lemniscus, and inferior colliculus in VPA exposed animals [13–16]. These neural changes are likely to cause disruption in the subcortical processing of sounds, but the existence and nature of any subcortical changes in physiology have not yet been characterized. Therefore, in this study, we tested the hypothesis that prenatal exposure to VPA degrades neural responses to sounds in a subcortical region of auditory processing, the inferior colliculus (IC).

## Methods

Inferior colliculus (IC) responses were recorded from two groups of rats: 1) saline-exposed rats (n = 19), and 2) VPA-exposed rats (n = 18). The University of Texas at Dallas Institutional Animal Care and Use Committee approved all surgical protocols and recording procedures.

### Subjects

Male and female Sprague Dawley rats were obtained from Charles River Laboratory (Wilmington, MA) and mated. Pregnancy was determined by the presence of a vaginal plug. On embryonic day 12.5 of pregnancy, the female rats received a single intraperitoneal injection of either 600 mg/kg of valproic acid (VPA) dissolved in 0.9% saline, or 0.9% saline alone, as in previous studies [10, 11, 17–19]. Thirty-seven male and female animals from ten litters were used for this study. Of the thirty-seven rats, 9 females and 10 males were used in the saline group, and 8 females and 10 males were used in the VPA group.

### Electrophysiology Recording

Multi-unit inferior colliculus responses were recorded from 490 sites in the saline-exposed control group, and 415 sites in the VPA-exposed group. All recordings were obtained in adult rats over 90 days of age. The rats were anesthetized with pentobarbital (50 mg/kg), and booster pentobarbital (8 mg/kg) was administered as needed. To ease the animal’s breathing, a tracheotomy was performed to directly deliver humidified air. Following the tracheotomy, a hole was made 9 mm posterior and 1.5 mm lateral to bregma over the right inferior colliculus. Two Parylene-coated tungsten microelectrodes (1–2 MΩ, FHC) were lowered to 1,000 microns below the pial surface and recordings were made at 200-micron intervals along the dorsal-ventral axis until a depth of 5,000 microns. At each 200 micron recording interval, auditory stimuli were presented through a Tucker Davis Technologies (TDT) MF-1 speaker that was placed 10 cm away from the left ear. The stimulus set at each recording site consisted of 1,296 tones of different frequency and intensity combinations from 1 to 32 kHz in 0.0625 octave steps and 0 to 75 dB SPL in 5 dB steps that were randomly interleaved. Additionally, 20 repeats of each of 15 randomly interleaved speech sounds (consonant-vowel-consonant) that differed by the initial consonant or the vowel were presented (‘chad’, ‘dad’, ‘deed’, ‘dood’, ‘fad’, ‘gad’, ‘had’, ‘jad’, ‘sad’, ‘shad’, ‘tad’). All speech sounds were presented so that the loudest 100 ms of the vowel portion of the sound was 60 dB. Additionally, the sounds ‘dad’ and ‘shad’ were also presented at 45 dB and 75 dB. Finally, 20 repeats of a noise burst train consisting of 6 25-ms noise bursts at a rate of 10 Hz were presented.

### Statistical Analysis

All data were analyzed using MATLAB software and SPSS version 27. The response strength evoked by speech sounds was calculated by taking the average number of driven spikes during the entire 400 ms duration of the sound, during the first 40 ms onset response to the consonant portion of the sound, and during the 300 ms response to the vowel portion of the sound. The consonants were sub-divided based on differences in manner of articulation (stop consonants, affricates, and fricatives) [20]. Onset latency was determined as the first spike latency after the presentation of each sound, and the peak latency was defined as the maximum firing rate latency [11, 21].

A nearest neighbor classifier was used to determine the neural discrimination accuracy between pairs of sounds by utilizing spike timing from single trial responses to each of the speech stimuli presented [11, 21]. This classifier determined the similarity of single trial responses to peri-stimulus time histogram (PSTH) templates generated from the remaining 19 repeats of the IC response that was evoked by each sound. The nearest-neighbor classifier identifies the sound most likely to have generated the single trial response under consideration using Euclidean distance. The onset response for all speech sounds was the initial 40 ms response to the consonant portion of the sound, binned using 1 ms precision. The consonant pairs included all speech sounds that differed in the initial consonant, with particular focus on consonant pairs differing within manner of articulation categories. For the vowel response, the classifier was provided with the vowel portion of the response, from 140 to 440 ms, using a single 300 bin [22]. The vowel pairs were ‘Dad-Deed’, ‘Dad-Dood’, and ‘Deed-Dood’. Classifier accuracy was quantified as the percentage of neural responses that were accurately assigned to the sound that generated the response.

Responses to tones at each IC recording site were also quantified. Receptive field properties (characteristic frequency, response threshold, bandwidth, response latencies, and spontaneous firing) in the inferior colliculus were calculated [23, 24]. Firing rate and vector strength were quantified for the responses to the noise burst trains. The firing rate from all 6 bursts was quantified by taking the average number of driven spikes. Vector strength was used to quantify the synchrony of the noise bursts [25]. For all analysis, normal distributions were determined using the Lilliefors Test for Normality. When the population was normally distributed, a generalized linear mixed model (GLMM) with Bonferroni correction was used when multiple recording sites were nested within individual rats or a two-way ANOVA was used when a single value was obtained for each rat to compare group responses. For the GLMM, the experimental group (VPA vs. saline) was evaluated as a fixed factor, and rat was evaluated as a random factor. Otherwise, non-parametric Mann-Whitney U statistics were used to compare group inferior colliculus responses to tones, speech, and noise bursts [26]. Ninety-five percent bootstrap confidence intervals of the median were computed using 50,000 samples.

## Results

### IC responses to speech sounds are altered in VPA-exposed rats

**Figure 1:**
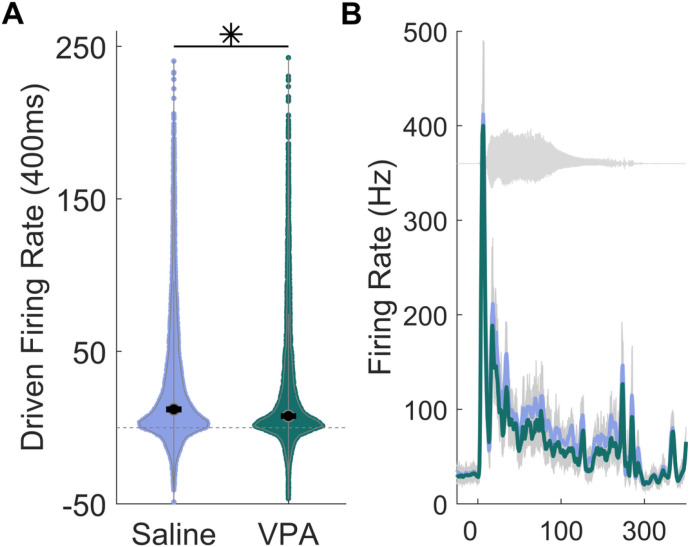
The IC response strength to speech sounds is weaker in VPA-exposed rats compared to saline-exposed rats. **A)** Violin plots showing the number of driven spikes evoked at each IC recording site for each speech sound. The driven rate was quantified using the 400 ms duration of the response. The black circle indicates the median, and error bars indicate the 95% confidence interval. The asterisk indicates experimental groups that are statistically significant from each other using a Mann-Whitney U test. **B)** Post-stimulus time histogram showing the IC response to the sound ‘dad’ presented at 60 dB. The sound waveform is plotted behind the PSTH in gray.

Delayed and weaker responses to sounds are commonly observed in both children and animal models of autism [1, 27–29]. VPA-exposed rats have also shown abnormal neural activity and degraded auditory cortical processing [10–12]. In our current study, we aimed to determine whether prenatal exposure to VPA could alter auditory responses in the IC. To do so, we first compared the IC responses to speech sounds between VPA-exposed rats and saline-exposed rats. Consistent with previous research [28, 30], we found that VPA-exposed rats had significantly weaker IC driven responses to speech sounds than saline-exposed rats (Mann Whitney U, p < 0.0001, Fig. 1a and b, Additional File 1).

**Figure 2:**
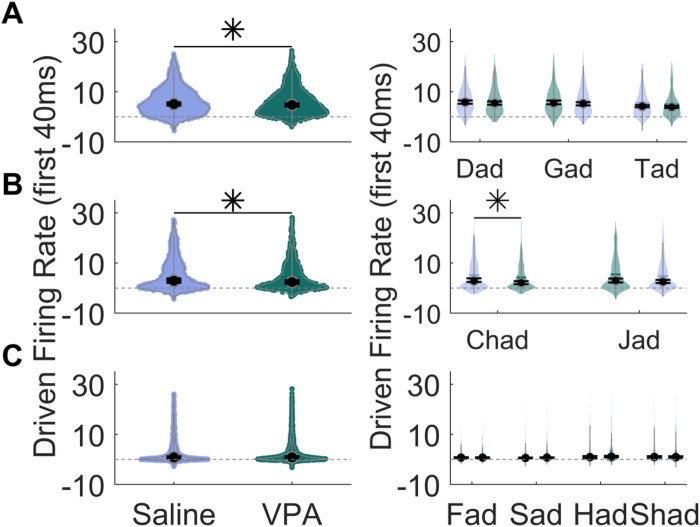
The IC response strength to the consonant portion of the speech sounds is altered in VPA-exposed rats compared to saline-exposed rats. **A)** Left, Violin plots showing the number of driven spikes evoked at each IC recording site for the consonant portion of each stop consonant sound. The driven rate was quantified using the 40 ms response to the consonant portion of the sounds. The black circle indicates the median, and error bars indicate the 95% confidence interval. Right, Violin plots depicting the average group response strength evoked by the consonants ‘d’, ‘g’, and ‘t’ presented at 60 dB. The asterisk indicates experimental groups that are statistically significant from each other using a Mann-Whitney U test. **B)** Left, Violin plots showing the number of driven spikes evoked at each IC recording site for the consonant portion of each affricate sound. Right, Violin plots depicting the average group response strength evoked by the consonants ‘ch’, and ‘j’ presented at 60 dB. **C)** Left, Violin plots showing the number of driven spikes evoked at each IC recording site for the consonant portion of each fricative sound. The driven rate was quantified using the 40 ms response to the consonant portion of the sounds. Right, Violin plots depicting the average group response strength evoked by the consonants ‘f’, ‘h’, ‘s’, and ‘sh’ presented at 60 dB.

The speech sounds were presented as consonant-vowel-consonant words, and varied either in the initial consonant or in the vowel portion of the word (i.e. ‘Dad’ vs ‘Gad’ or ‘Dad’ vs ‘Deed’). To determine whether VPA-exposed rats had deficits to specific portions of the sound, we compared responses between groups to both the consonant portion of the sounds and the vowel portion of the sounds. For consonant sounds, we first examined the IC response to stop consonants (‘d’, ‘g’, ‘t’), that are known to have rapid spectrotemporal transitions. There was a significant difference between groups in the response strength to the consonant portion of the sounds for stop consonants (Mann Whitney U, p = 0.034, Fig. 2a). We next examined the IC response to affricate sounds (‘ch’ and ‘j’), which transition from stop consonant-like acoustics to fricative-like sounds, with a more rapid fricative portion of the sound compared to a pure fricative [20]. There was a significant difference between groups in the response strength to the consonant portion of the sounds for affricate sounds (Mann Whitney U, p = 0.03, Fig. 2b). Finally, we examined the IC response to fricative sounds (‘f’, ‘h’, ‘s’, and ‘sh’), which involve a partial obstruction of the vocal tract. Interestingly, there was no significant alterations between groups in the response strength to the consonant portion of the sounds for fricative sounds (Mann Whitney U, p = 0.404, Fig. 2c). These results indicate that the spectrotemporal acoustics of the consonant are an important factor in the IC response strength to consonant sounds.

**Figure 3:**
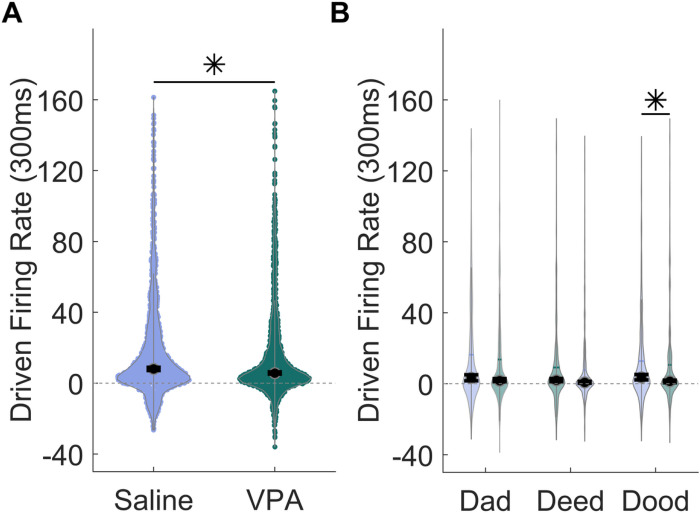
The IC response strength to the vowel portion of the speech sounds is altered in VPA-exposed rats compared to saline-exposed rats. **A)** Violin plots showing the number of driven spikes evoked at each IC recording site for the vowel portion of each speech sound. The driven rate was quantified using the 300 ms response to the vowel portion of the sounds. The black circle indicates the median, and error bars indicate the 95% confidence interval. The asterisk indicates experimental groups that are statistically significant from each other using a Mann-Whitney U test. **B)** Violin plots depicting the average group response strength evoked by the vowels ‘a’, ‘ee’, and ‘oo’ presented at 60 dB.

Similarly, the response strength to the vowel portion of the sounds was significantly altered, with VPA-exposed rats responding significantly weaker to the vowel portion of the sound compared to saline-exposed rats (Mann Whitney U, p = 0.01, Fig. 3). Responses to both the consonant onset as well as the sustained vowel portions of the sound were significantly weaker in VPA-exposed rats compared to saline-exposed rats.

**Figure 4:**
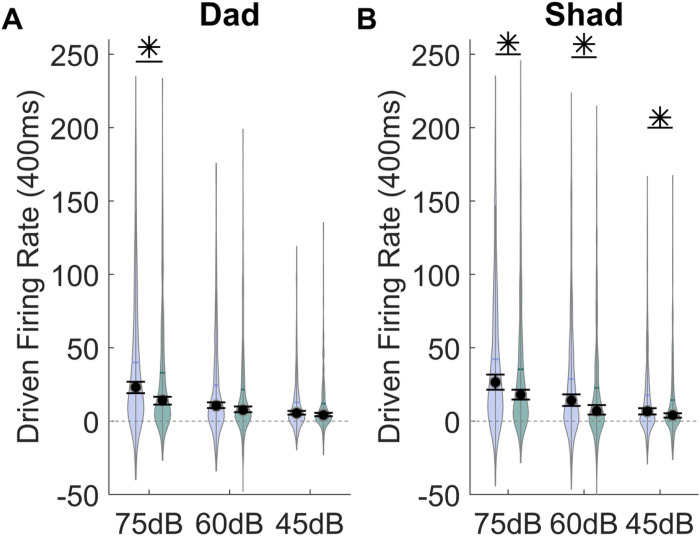
The IC response strength to speech sounds is altered over multiple sound intensities in VPA-exposed rats compared to saline-exposed rats. **A)** Violin plots depicting the average group response strength evoked by the sound ‘dad’ presented at 75, 60, and 45 dB. The driven rate was quantified using the 400 ms response to the sounds. The black circle indicates the median, and error bars indicate the 95% confidence interval. The asterisk indicates experimental groups that are statistically significant from each other using a Mann-Whitney U test. **B)** Violin plots depicting the average group response strength evoked by the sound ‘shad’ presented at 75, 60, and 45 dB.

In addition to presenting sounds differing in consonant or vowel, the sounds ‘Dad’ and ‘Shad’ were presented at three different intensities (75, 60, 45 dB). VPA-exposed rats had a significantly weaker response strength evoked by ‘dad’ presented at multiple sound intensities compared to the saline-exposed rats (Mann Whitney U, p = 0.004, Fig. 4a). The sound ‘dad’ presented at 75 dB showed the greatest difference in response strength between the groups (Mann Whitney U, p = 0.02). VPA-exposed rats also had a significantly weaker response strength evoked by ‘shad’ presented at multiple sound intensities compared to the saline-exposed rats (Mann Whitney U, p < 0.0001, Fig. 4b). The sound ‘shad’ presented at 60 and 75 dB showed the greatest difference in response strength between the groups (Mann Whitney U, p = 0.02). These results suggest that VPA-exposed rats have a greater response strength deficit to louder sounds compared to quieter sounds.

**Figure 5:**
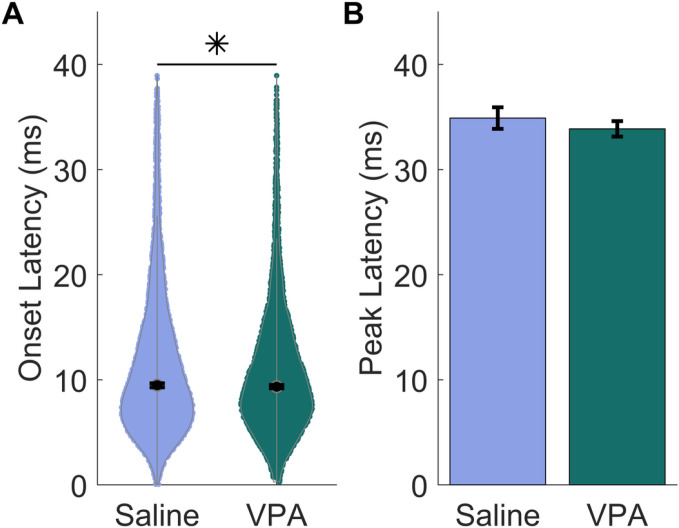
The speech response timing is significantly faster in VPA-exposed rats than saline-exposed rats. **A)** Violin plots showing the timing of the onset response latency to speech sounds. The saline-exposed rats were significantly slower than the VPA-exposed rats. The black circle indicates the median, and error bars indicate the 95% confidence interval. **B)** Bar plot comparing the timing of the peak response latency to speech sounds. There was no significant difference in the peak latency between VPA-exposed rats and saline-exposed rats.

In addition to response strength changes, differences in the timing of responses to speech have been reported in individuals with autism and animal models of ASD [11, 21, 29, 30]. In this study, there was a significant difference in the onset latency, but not in peak latency between the experimental groups. Contrary to the previous findings, the saline-exposed rats had a significantly slower onset response latency to speech sounds than the VPA-exposed rats, while the peak response latency was unaltered between groups (Mann Whitney U, Onset: p = 0.027; GLMM, Peak: F(1,903) = 0.65, p = 0.42; Fig. 5a & b). Overall, our results suggest that VPA-exposed rats have altered responses to speech sounds.

### IC responses to tones are unaltered in VPA-exposed rats

**Figure 6:**
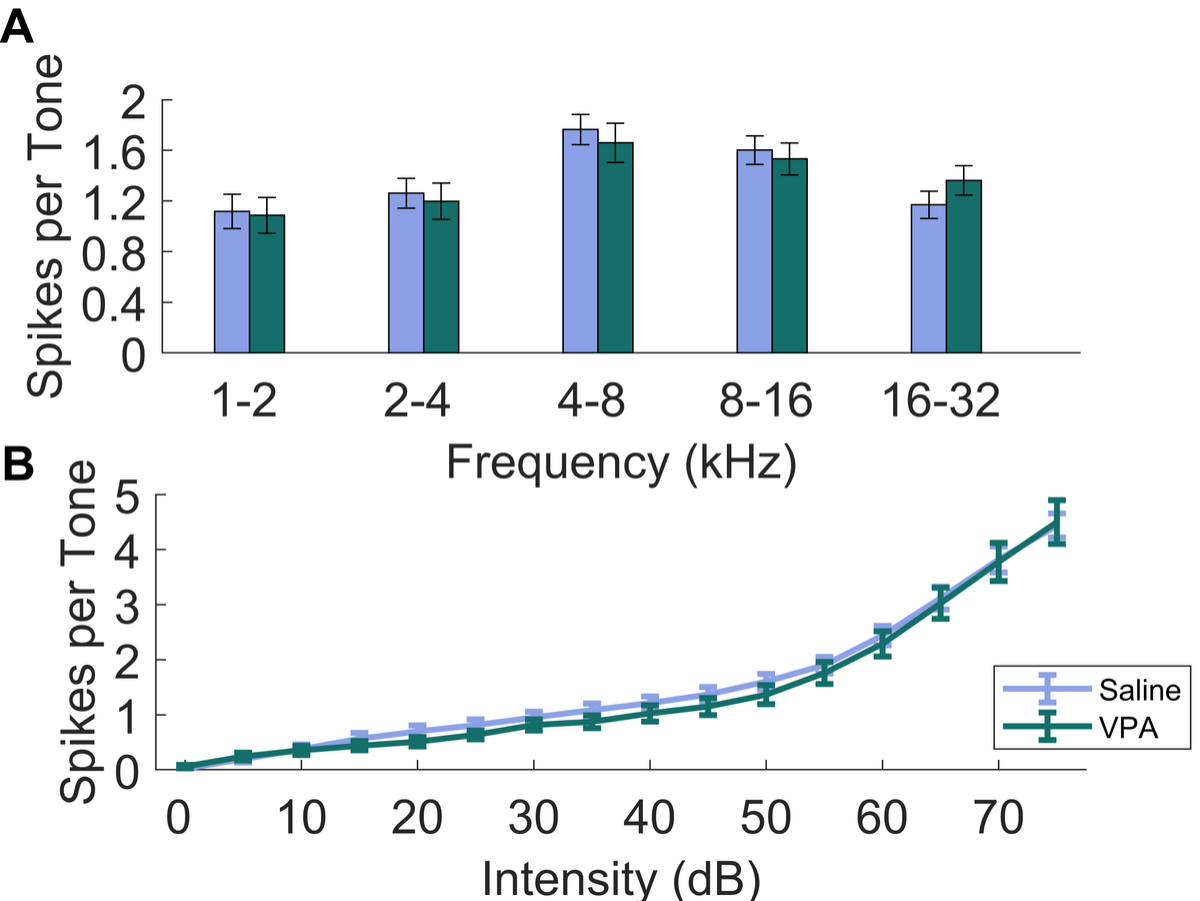
The IC response to pure tones of 1–32 kHz and 0–75 dB were unaltered in VPA-exposed rats. **A)** Bar plots showing the average number of spikes across tone frequency, binned in 1 octave bins. The error bars are standard error of the mean across rats. There were no significant differences in the response strength comparing saline-exposed rats to VPA-exposed rats across frequency bins. **B)** Rate-intensity function comparing the number of spikes evoked per tone based on sound intensity. The error bars represent standard error of the mean across rats. For both groups, as the sound intensity increased, there was an increase in the number of evoked spikes. However, no significant differences were found between groups across each intensity.

In addition to speech sounds, tones consisting of frequencies from 1–32 kHz and intensities from 0–75 dB were presented to investigate alterations in tonotopic organization and receptive field properties in the inferior colliculus. To investigate whether the VPA rats exhibit alterations in the response to tones in the IC, the number of spikes tuned to multiple frequency ranges were compared between the two groups. There were no significant differences between VPA-exposed rats and saline-exposed rats (Two-way ANOVA, F(1,175) = 0.037, p = 0.848, Fig. 6a). Next, the rate-intensity function to tones was examined to quantify the response to tones at multiple sound intensity levels. As expected, for both groups, increasing tone intensity evoked stronger IC responses (Two-way ANOVA, F(15,560) = 124.26, p < 0.01). Comparing saline-exposed and VPA-exposed rats, there were no significant differences between experimental groups across intensities (Two-way ANOVA, F(1,560) = 2.022, p = 0.156, Fig. 6b).

Additionally, receptive field properties including response threshold, bandwidths at 10 and 40 dB above the threshold, onset and peak latency, end of peak latency, and spontaneous firing rates were examined between groups. There were no significant differences between the groups for any receptive field properties (Threshold: F(1,34) = 0.97, p = 0.33; Bandwidth at 10 dB: F(1,35) = 3.23, p = 0.08; Bandwidth at 40 dB: F(1,35) = 1.17, p = 0.29; Onset Latency: p = 0.72; Peak Latency: p = 0.59; End of Peak Latency: p = 0.27; and Spontaneous Firing Rate: p = 0.95, Table 1). Overall, VPA-exposed rats did not show significant differences in their IC responses to tones compared to saline-exposed rats.

### IC temporal processing is intact in VPA-exposed rats

**Figure 7:**
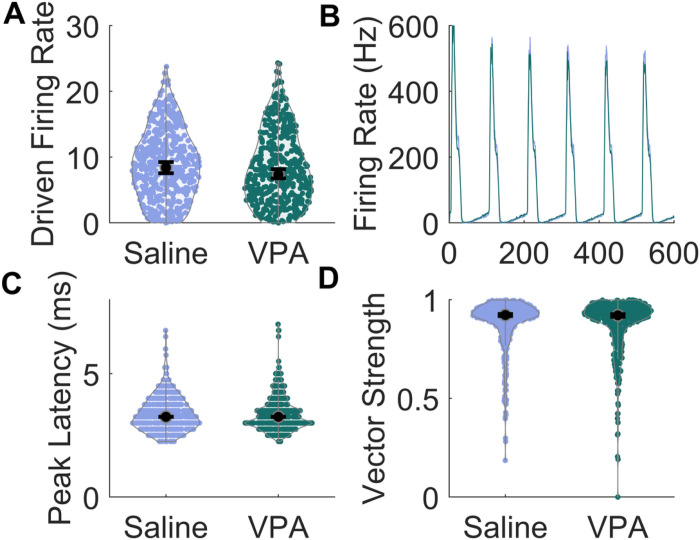
Responses to 10 Hz trains of noise bursts were compared between the groups. **A)** The response strength evoked by noise burst trains showed no alterations in VPA-exposed rats. The black circle indicates the median, and error bars indicate the 95% confidence interval. **B)** Average peristimulus time histogram (PSTH) to noise bursts presented six times at 10 Hz. **C)** Peak latency response to the first noise burst in saline-exposed and VPA-exposed rats. No significant differences were observed between groups. **D)** The vector strength was unaltered in VPA-exposed rats compared to saline-exposed rats.

In both humans and animal models of autism, the cortical phase locking to rapidly presented sounds is often impaired, with both a decreased neural amplitude and less synchronous responses evoked by sounds [1, 11, 30, 31]. To investigate whether this is also observed in the inferior colliculus, we presented 10 Hz trains of noise bursts. As seen with responses to tones, there was no significant difference in the peak firing rate evoked by noise burst trains between the saline-exposed rats and the VPA-exposed rats (p = 0.18; Fig. 7a & b). In addition, the timing of the first peak of the response was not significantly different between the groups (p = 0.88; Fig. 7c). To quantify synchrony, the vector strength was examined. The vector strength was also unaltered between the two groups (p = 0.67; Fig. 7d). These results suggest that, in the IC, exposure to VPA does not influence temporal processing, at a relatively slow rate of 10 Hz.

### IC neural discrimination accuracy is degraded in VPA-exposed rats

A nearest-neighbor classifier was used to quantify the neural discrimination of pairs of speech sounds. A previous study found that in the anterior auditory field, VPA-exposed rats had significantly worse discrimination accuracy of consonant pairs, but not vowel pairs, compared to saline-exposed rats [11]. In our current study, the discriminability of IC responses to pairs of speech sounds were compared. Overall, the IC neurons of the VPA-exposed rats were significantly less accurate at discriminating stop consonant pairs (D-G, D-T, G-T) compared to saline-exposed rats (Mann Whitney U, p = 0.0071; Table 2). Surprisingly, the VPA exposed rats more accurately discriminate stop consonants at recording sites with low frequency CFs compared to saline exposed rats (Mann Whitney U, p = 0.0031). Meanwhile at recording sites tuned to high frequencies, the VPA-exposed rats were significantly less accurate than saline-exposed rats (Mann Whitney U, p < 0.01). We next examined the affricates and the fricatives. For both, the VPA-exposed rats were worse at discriminating affricate and fricative pairs of sounds at high frequency recording sites (Mann Whitney U, Affricates: p = 0.0013; Fricatives: p < 0.01). Discriminating affricate and fricative pairs of sounds at low frequency recordings sites were not significantly different (Mann Whitney U, Affricates: p = 0.07, Fricatives: p = 0.22). Comparing neural discrimination to vowel pairs, VPA-exposed rats were significantly worse than saline-exposed rats at high frequency sites (p < 0.01). These results indicate that neural responses to unique speech sounds evoke more similar neural activity patterns in VPA-exposed rats and more distinct neural activity patterns in saline-exposed rats.

## Discussion

Individuals with ASD have auditory brainstem response (ABR) abnormalities, which presents as delayed and weaker brainstem responses to sounds compared to neurotypical individuals [30, 31]. Previous studies have observed anatomical differences along the auditory pathway in rodents prenatally exposed to VPA [11, 13, 15, 31, 32]. The purpose of this study was to investigate subcortical auditory processing physiology in prenatally exposed VPA rats. This study expands on previous studies that have documented that prenatal exposure to VPA affects auditory processing in both cortical and subcortical regions [1, 13, 33]. In this study, VPA exposed rats had significantly weaker and delayed responses to speech sounds compared to saline-exposed control rats in the inferior colliculus. Meanwhile, VPA-exposed rats had no alterations in the response to tones and noise bursts compared to saline-exposed control rats. These results suggest that VPA-exposed rats have intact IC processing of simple sounds but are impaired at processing complex stimuli.

### Complex versus simple stimuli processing in ASD

Children with ASD have difficulty processing spectrotemporally complex sounds, like speech sounds, to a greater extent than simple sounds, like tones [1, 29, 34–36]. These children have significantly weaker and delayed responses to complex sounds than they do simple sounds when compared to typically-developing children [1, 28, 31, 35, 37–40]. For example, one study found that when children with autism were presented with the speech sound /da/, these children had significantly weaker ABR responses compared to typically developing children [41]. Our current study supports the previous findings; the VPA-exposed rats displayed significantly degraded IC responses to speech sounds compared to saline-exposed rats, but their responses to tones were intact. Previously, VPA-rats were found to also process speech sounds significantly weaker and slower in the anterior auditory field (AAF) compared to saline exposed rats [33]. In addition to the abnormal cortical and subcortical auditory processing observed in these rats, VPA rats also perform consonant discrimination tasks, but not vowel discrimination tasks, with significantly less accuracy than the saline-exposed control rats [10]. These findings together provide further evidence that VPA exposure affects the processing of complex sound stimuli. This disrupted auditory processing could potentially contribute to the receptive language and social communication impairments that are commonly observed in children with ASD [9, 34].

### The auditory pathway in VPA-exposed rodents

Previous studies have found that VPA exposed rats have degraded processing of sounds in the primary auditory cortex and the anterior auditory field (AAF) [11, 12]. While no studies have documented neuron spiking activity subcortically in VPA-exposed rodents, multiple papers document that there is abnormal neural structure subcortically in VPA-exposed rodents. VPA-exposed rodents have fewer neurons in the ventral cochlear nucleus, superior olivary complex, inferior colliculus, and medical geniculate nucleus of the thalamus [16, 42]. Interestingly, a recent paper exhibited elevated ABR thresholds and increased latencies to clicks in VPA-exposed animals at 22 days of age, but not 60 days of age [15]. This fascinating finding suggests that there may be a delayed maturation of auditory brainstem responses to simple sounds, like clicks, and provides hope that the severely impacted responses observed in response to more complex sounds, like speech, could potentially improve with age.

### The auditory pathway in children with ASD

Previous studies have found alterations in the neural response to sounds across the auditory pathway in individuals with ASD. Subcortically, individuals with ASD have been documented to have normal click-evoked brainstem responses, but neural synchrony and phase locking brainstem response deficits to speech sounds [30, 31]. Responses in primary auditory cortex were significantly delayed in children with both ASD and hypersensitivity compared to children with ASD without hypersensitivity and to typically developing children [43]. Interestingly, another study has documented unaltered speech-evoked responses in primary auditory cortex, but reduced speech-evoked responses in the superior temporal gyrus (STG) in individuals with ASD [44]. Response latencies are also greatly degraded in individuals with ASD in the STG. Compared to typically developing children, verbal children with ASD have significantly longer STG latencies to vowel sounds, language impaired children with ASD have latencies that are further delayed, and minimally-verbal/non-verbal children with ASD have profoundly delayed latencies to vowel sounds in the STG [3]. Overall, children with ASD often exhibit degraded neural responses to speech sounds across the auditory pathway.

### Future studies

The current study showed VPA-exposed rats have degraded responses to speech sounds in the inferior colliculus, specifically to stop consonants, affricates, and vowels. The neural classifier also revealed that VPA-exposed rats are worse at discriminating consonants and vowels compared to saline-exposed rats. This finding is consistent with previous studies, as Engineer et al. (2014) found VPA-exposed rats performed worse at consonant discrimination tasks compared to saline-exposed rats. Extensive auditory discrimination training improved the responses in the anterior auditory field in trained VPA-exposed rats compared with untrained VPA-exposed rats. Therefore, it may also be beneficial to document inferior colliculus responses after extensive auditory discrimination training.

Furthermore, the usage of vagus nerve stimulation (VNS) paired with behavioral training may improve auditory processing in both animal models and children with autism [45, 46]. Previous studies have found that vagus nerve stimulation significantly improves the frequency and severity of seizures, which are common co-morbid disorders in children with autism [47–51]. Additionally, pairing VNS with sounds significantly strengthens responses in typically hearing rats across the auditory pathway [23, 52]. VNS has also been paired with tones in a rat autism model of Rett syndrome, heterozygous *Mecp2* rats [21]. Pairing VNS with tones for 20 days significantly improved the primary auditory cortex response to sounds in *Mecp2* rats. Future studies pairing VNS with sounds are needed to document both behavioral discrimination ability and auditory responses along the auditory pathway in multiple rat models of ASD.

## Figures and Tables

**Table 1:** Receptive field properties based on responses to tones compared between saline-exposed and VPA-exposed rats. Threshold, bandwidth at 10 dB, and bandwidth at 40dB were normally distributed and a generalized linear mixed model was used for comparison. Latency, peak latency, end of peak, and spontaneous were not normally distributed, so groups were compared with a Mann Whitney U. Overall, no significant differences were observed between groups.

	Saline		VPA	
	Mean	Median (95% CI)	Mean	Median (95% CI)
Threshold (dB)	20.79	20.01 (15.89–25.57)	21.07	20.7 (17.09–25.93)
Bandwidth 10 dB (octaves)	1.46	1.46 (1.13–1.64)	1.71	1.70 (1.30–1.90)
Bandwidth 40 dB (octaves)	1.61	1.33 (1.02–1.82)	1.74	1.81 (1.20–2.36)
Latency (ms)	8.19	8.07 (7.62–8.61)	8.01	8.15 (7.48–9.14)
Peak Latency (ms)	13.34	12.88 (12.35–13.93)	12.48	12.70 (11.62–14.35)
End of Peak (ms)	27.10	27.17 (26.32–27.56)	25.52	26.26 (25.23–27.01)
Spontaneous (Hz)	32.55	29.35 (8.89–37.39)	30.58	21.75 (14.65–36.07)

**Table 2:** Neural classifier accuracy based on both the consonant portion of the sound (40 1-ms bins), organized into three different phonetic groups, and the vowel portion of the sound (1 300-ms bin). The numbers represented in this table are shown as median (95% confidence interval). The bolded numbers indicate experimental groups that are statistically significant from each other across low and high frequency recording sites using a Mann-Whitney U test. Classifier accuracy was determined for speech sound pairs for low frequency neurons (1–8 kHz) and high frequency neurons (9–32 kHz).

		Low Frequency (1–8 kHz)		High Frequency (9–32 kHz)	
		Saline	VPA	Saline	VPA
**Consonant**	**Stops (D/G/T)**	70 (67.5–72.5)	**77.5 (75–82.5)**	85 (82.5–87.5)	**72.5 (67.5–75)**
	**Affricates (Ch/J)**	95 (92.5–95)	92.5 (90–95)	95 (92.5–97.5)	**92.5 (90–95)**
	**Fricatives (F/H/S/Sh)**	95 (92.5–95)	92.5 (92.5–95)	97.5 (97.5–97.5)	**95 (95–97.5)**
**Vowels**	**(-ad/-eed/-ood)**	80 (75–82.5)	77.5 (75–81.25)	80 (77.5–82.5)	**72.5 (70–75)**

## Data Availability

All datasets and analysis code are available upon request to the corresponding author.

## Abbreviations

ASD: Autism spectrum disorders
VPA: Valproic acid
IC: Inferior colliculus
AAF: Anterior auditory field
STG: Superior temporal gyrus
VNS: Vagus nerve stimulation
